# Cognitive function and speech outcomes after cochlear implantation in older adults

**DOI:** 10.3389/fneur.2025.1630946

**Published:** 2025-07-10

**Authors:** Tadao Yoshida, Masumi Kobayashi, Daisuke Hara, Rikako Taniguchi, Yukari Fukunaga, Michihiko Sone

**Affiliations:** ^1^Department of Otorhinolaryngology, Nagoya University Graduate School of Medicine, Nagoya, Japan; ^2^Department of Rehabilitation, Nagoya University Graduate School of Medicine, Nagoya, Japan

**Keywords:** cochlear implantation, cognitive function, speech perception, hearing loss, auditory

## Abstract

**Background:**

The impact of cochlear implantation on cognitive function in older adults and the relationship between preoperative cognitive ability and postoperative speech perception remain poorly understood. In this study, we aimed to evaluate the effect of cochlear implant use on cognitive function in older adults and to explore the association between preoperative cognitive ability and postoperative speech discrimination.

**Methods:**

We conducted a retrospective cohort study at a university hospital between June 2017 and March 2025. Thirty cochlear implant recipients aged ≥61 years were included, with 21 receiving unilateral implants and nine receiving bilateral implants. All participants underwent cognitive assessments both preoperatively and postoperatively. We analyzed the cognitive function test results before and after cochlear implantation. The primary outcomes measured were: (1) the correlation between preoperative cognitive test scores and postoperative speech discrimination scores; and (2) longitudinal changes in postoperative cognitive function.

**Results:**

A significant positive correlation was observed between preoperative Kohs Block Design Test scores and postoperative speech discrimination scores (*p* < 0.01). Preoperative Raven’s Colored Progressive Matrices scores also correlated positively with postoperative speech discrimination scores (*p* < 0.05). Postoperatively, Kohs scores demonstrated significant positive correlations with both the Mini-Mental State Examination (*p* < 0.01) and Reading Cognitive Test Kyoto test (*p* < 0.0001) scores. Following a 3.7-year mean follow-up, Kohs scores remained stable, with some patients showing improvements.

**Conclusion:**

Cognitive assessments performed during the preoperative CI evaluation may yield valuable insights into postoperative outcomes in older adults. Additionally, long-term postoperative cognitive function is generally preserved, with the potential for improvement following cochlear implantation.

## 1 Introduction

Hearing loss is a significant global health issue, affecting over 5% of the world’s population (approximately 430 million people). By 2050, this number is estimated to exceed 700 million, or one in 10 individuals. The prevalence of hearing loss increases with age; among individuals older than 60 years, more than 25% experience disabling hearing loss (1). Regardless of severity, untreated hearing loss has substantial implications for communication, social interaction, and cognitive function, particularly in older adults (2, 3). Recent studies have highlighted the association between hearing loss and cognitive decline in aging populations (4, 5). Age-related hearing loss has been identified as a modifiable risk factor for dementia, and evidence suggests that auditory deprivation accelerates neurocognitive decline (6, 7). Several mechanisms have been proposed to explain the association between hearing loss and cognitive decline, including increased cognitive load, auditory deprivation, and reduced social engagement. While epidemiological studies consistently show that hearing loss is associated with an elevated risk of cognitive impairment and dementia, the causal mechanisms remain unclear (4–7). In particular, whether hearing rehabilitation, such as cochlear implantation, can slow or reverse cognitive decline in older adults remains unclear.

The cognitive load hypothesis has been proposed to explain this association (8). The concept of cognitive load in individuals with hearing loss is critical for evaluating the impact of auditory impairment on information processing and cognitive function. Individuals with hearing loss require significantly more cognitive resources to recognize speech and language than those with normal hearing (9). This increased cognitive load may affect other cognitive processes, including attention, memory, learning, and decision-making (10, 11). When individuals with hearing loss encounter difficulty understanding speech, the brain compensates by working harder to fill in the missing auditory information – a phenomenon referred to as “effortful listening” (12). This reduces the cognitive resources available for other tasks. Additionally, when auditory information is incomplete, individuals often rely on visual cues, such as lip reading, gestures, and facial expressions, which further increase the cognitive load in the visual domain (13). Prolonged exposure to a high cognitive load can lead to cognitive fatigue and depletion of cognitive resources in individuals with hearing loss, potentially resulting in brain degeneration and atrophy (9, 14). Severe hearing loss has been reported to be associated with an increased risk of cognitive impairment, with evidence of a dose–response relationship between the degree of hearing loss and dementia risk (15).

Another factor that may influence long-term speech perception outcomes in older adult cochlear implant users is age-related degeneration of both the peripheral and central auditory systems (16). Sladen et al. reported that unilateral cochlear implant users (*n* = 21, mean age = 77 years) experienced benefits in speech understanding in both quiet and noisy conditions, as well as improvements in quality of life (17). Furthermore, among cochlear implant users aged 65 years and above, consonant-nucleus-consonant word scores remained stable between 6 months and 1 year post-implantation. Notably, they showed significant improvement between 1 and 5 years, followed by stabilization between 5 and 10 years. A similar trend was observed in noise test (HINTS) sentence scores, which remained stable between 6 months and 1 year and between 5 and 10 years while showing significant improvement in the later years (18). These findings suggest that cochlear implant use contributes to long-term stability in speech and auditory performance (19, 20).

However, research on the effect of cochlear implants on cognitive function remains limited, particularly regarding their effectiveness in older adults (21). The primary objective of this study was to examine the relationship between cognitive function and auditory outcomes in older adults following cochlear implantation. Specifically, we aimed to investigate whether preoperative cognitive performance predicts postoperative speech perception outcomes, and to assess changes in cognitive function after implantation as a secondary objective. We hypothesized that the findings of this study would contribute to the development of improved treatment strategies for age-related hearing loss and enhance the quality of life for older individuals.

## 2 Materials and methods

We included 30 patients (19 females and 11 males) aged 61–86 years (mean age: 73.8 years), who underwent cochlear implantation (CI) and were evaluated postoperatively between 2017 and 2023. Nine patients received bilateral implants. The etiology of hearing loss on the implanted side was idiopathic in 26 patients, chronic otitis media in two, sudden sensorineural hearing loss in one, and drug-induced hearing loss in one. Twenty-one patients underwent unilateral implantation (13 left and 8 right), while nine received bilateral implants, including six who underwent simultaneous bilateral procedures. The age at implantation ranged from 58 to 83 years (mean [standard deviation], 70.1 [6.1] years), with a mean duration of deafness of 5.8 years (range, 0.5–56 years). Devices were manufactured by Cochlear Corporation (*n* = 23) and MED-EL Corporation (*n* = 7), including 7 and 2 bilateral users, respectively. All surgeries were performed between June 2017 and September 2023, ensuring comparable generations of electrode arrays.

To reflect the diversity of clinical practice, we adopted broad inclusion criteria encompassing a heterogeneous group of older adult CI users. Variability in CI usage duration, timing of cognitive and audiological assessments, and implant laterality was accepted to enhance the generalizability of findings. This observational design aimed to capture a wide range of real-world experiences, rather than to isolate the effects of specific variables.

No structured postoperative auditory or cognitive rehabilitation programs were implemented. All patients underwent standard clinical follow-up, which included device programming and general counseling on CI use. Some individuals may have engaged in self-directed auditory or cognitive activities; however, these were not systematically documented. Consequently, any observed changes in cognitive function were attributed to cochlear implant use rather than targeted rehabilitative interventions.

Our study focused on speech discrimination testing in quiet conditions to establish a standardized and controlled baseline of auditory perception following CI. This approach allowed us to evaluate fundamental speech recognition abilities without the additional variability introduced by background noise, which can differentially affect individuals based on numerous cognitive and auditory factors.

Preoperative assessments comprised speech discrimination testing using hearing aids and cognitive evaluations with the Kohs Block Design Test (KOHS) and Raven’s Colored Progressive Matrices (RCPM). For individuals with profound hearing loss and no benefit from amplification, speech discrimination testing was conducted unaided.

Postoperative evaluations were conducted at an average of 3.7 years following CI (range, 1–8 years). Speech discrimination testing using the cochlear implant was performed in all 30 patients, while cognitive assessments, including the Mini-Mental State Examination (MMSE), reading cognitive data were available for 27 patients (KOHS and RCPM) and 24 patients (Reading Cognitive Test Kyoto (ReaCT Kyoto) and MMSE). Reasons for missing data included worsening of pre-existing medical conditions (*n* = 1), death (*n* = 1), and loss to follow-up (*n* = 4). These data were analyzed to evaluate the impact of cochlear implant use on auditory and cognitive performance.

This study was approved by the Ethics Review Committee of Nagoya University School of Medicine, Nagoya, Japan (No. 2025-0009). Informed consent was obtained from all individuals included in the study.

### 2.1 Speech discrimination test

Monosyllabic speech discrimination was evaluated in a quiet environment using the 67-S speech audiometric test developed by the Japan Audiological Society (22). This test includes 20 monosyllables presented at a sound pressure level of 40–50 dB above the hearing threshold. The speech recognition curve was determined by administering the test three to five times, with sound pressure levels increased in 10–15 dB increments. The maximum speech discrimination score (SDS) was defined as the highest score obtained across trials.

Testing adhered to ISO-8253-2 guidelines (23) and was performed in a quasi-free sound field with the speaker positioned at least 1 m from the examinee and aligned at ear level. Sound pressure level variations at the examinee’s head were controlled to ensure measurement accuracy. Preoperative testing was conducted using headphones.

### 2.2 Cognitive assessments

Cognitive function was evaluated using four validated instruments: the MMSE, ReaCT Kyoto, KOHS, and RCPM. These tools collectively assess domains including memory, attention, visuospatial processing, and abstract reasoning. Brief descriptions of each test are provided below.

#### 2.2.1 Mini-mental state examination

The MMSE is a widely used 30-point screening tool assessing orientation, memory, attention, language, and visuospatial skills (24). Scores ≤ 23 indicate cognitive impairment, while scores between 24 and 27 suggest mild cognitive impairment. Given the auditory-verbal nature of the test, post-CI improvements in hearing may influence MMSE outcomes.

#### 2.2.2 Reading cognitive test Kyoto

The ReaCT Kyoto is a recently developed, visually administered cognitive test designed to be independent of auditory input. Its validity and reliability have been demonstrated in prior studies (25). It exhibits a strong correlation with the Japanese MMSE (*r* = 0.904) and high internal consistency (Cronbach’s alpha coefficient [*α*] = 0.87). The total score is 40, with a cutoff of 31/32 indicating cognitive impairment. Its standardized format ensures test consistency regardless of examiner experience and is particularly suited for individuals with hearing impairment.

#### 2.2.3 Kohs block design test

The KOHS is a nonverbal performance-based intelligence test developed in 1920 by Samuel C. Kohs, comprising 16 colored blocks used to replicate geometric patterns (26). It assesses visuospatial reasoning, executive function, and constructional ability. Scoring incorporates accuracy, completion time, and movement, with a maximum score of 131. The KOHS is historically used in individuals with language or hearing impairments and is incorporated into broader cognitive batteries such as the Wechsler scales. The reference values for older adults in Japan are as follows: 58.4 ± 13.9 (SD) for those in their 60s, 57.0 ± 11.8 (SD) for those in their 70s, and 51.9 ± 11.8 (SD) for those in their 80s.

#### 2.2.4 Raven’s colored progressive matrices

The RCPM is a nonverbal assessment of fluid intelligence and visual pattern recognition (27). Participants select the correct image to complete a visual matrix, evaluating problem-solving and abstract reasoning abilities. It is widely applied in individuals with communication or hearing difficulties. The reference values for older adults in Japan are as follows: 29.2 ± 5.40 (SD) for those in their 60s, 26.9 ± 5.40 (SD) for those in their 70s, and 24.9 ± 5.27 (SD) for those in their 80s.

### 2.3 Statistical analyses

Statistical analyses were conducted using IBM SPSS Statistics (version 30; IBM Corp., Armonk, NY, USA). Spearman’s rank correlation coefficients were calculated to assess associations among variables, with statistical significance set at *p* < 0.05. Receiver operating characteristic curve analysis was employed to evaluate the predictive performance of KOHS and RCPM scores, with optimal cutoff values determined using the Youden index.

## 3 Results

Detailed patient characteristics are presented in Table 1. All patients met the CI candidacy criteria for implantation, including severe-to-profound sensorineural hearing loss and limited benefit from appropriately fitted hearing aids. The mean preoperative SDS, measured in the sound field using the 67-S test, was 14.8%. This improved to a mean of 58.7% postoperatively. In bilateral users, SDS was assessed using both implants simultaneously. Preoperative cognitive function scores averaged KOHS IQ = 81.9 and RCPM = 27.0 while corresponding postoperative scores were KOHS IQ = 83.2 and RCPM = 28.1 at a mean follow-up of 3.7 years (range, 1–8 years). These slight increases were not statistically significant (KOHS: *p* = 0.881; RCPM: *p* = 0.763). The mean postoperative MMSE and ReaCT Kyoto scores were 26.5 and 33.1, respectively. The mean postoperative SDS was significantly higher in the MED-EL group compared to the Cochlear group (*p* ≤ 0.05, t-test). Preoperative and postoperative KOHS IQ scores were 83.6 and 80.3 in the MED-EL group, and 81.4 and 84.2 in the Cochlear group, respectively. Similarly, RCPM scores were 27.6 preoperatively and 27.3 postoperatively in the MED-EL group, and 26.8 and 28.5 in the Cochlear group. No significant differences in cognitive outcomes were observed between the two groups (*p* ≥ 0.05 for both KOHS and RCPM, t-test).

**Table 1:** Characteristics of the patients.

	Preoperative	Postoperative
Sex (female: male)	19: 11	
Unilateral: bilateral CI	21: 9	
Duration of deafness (y)	5.8 (0.5–56)	
Age (y)	70.1 (58–83)	73.8 (61–86)
KOHS (IQ)	81.9 (46.9–124.8)	83.2 (32.8–124.8)
RCPM	27.0 (13–35)	28.1 (18–36)
MMSE-J		26.5 (13–30)
ReaCT Kyoto		33.1 (14–40)
SDS (%)	14.8 (0–55)	58.7 (15–95)

SDS, speech discrimination scores; CI, cochlear implants; KOHS IQ, Kohs block design test; RCPM, Raven’s colored progressive matrices; MMSE-J, Mini-Mental State Examination Japanese adaptation.

### 3.1 Preoperative and postoperative speech discrimination scores

**Figure 1:**
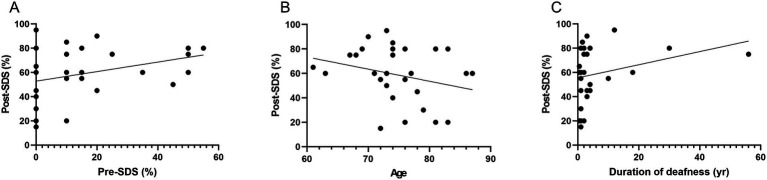
Association between preoperative and postoperative speech discrimination scores and related variables. **(A)** Preoperative SDS with hearing aids vs. postoperative SDS with CI: Scatter plot illustrating the relationship between preoperative SDS using hearing aids and postoperative SDS with CI. No significant correlation was observed (*p* = 0.08, *r* = 0.325), indicating limited predictive value of preoperative aided speech discrimination for postoperative performance. **(B)** Age at implantation vs. postoperative SDS: Scatter plot depicting the association between age at implantation and postoperative SDS. A non-significant negative correlation was found (*p* = 0.14, *r* = −0.271), suggesting a potential trend of reduced speech perception with increasing age. **(C)** Duration of deafness vs. postoperative SDS: Scatter plot evaluating the relationship between duration of deafness prior to implantation and postoperative SDS. No significant correlation was found (*p* = 0.139, *r* = 0.276), indicating that auditory deprivation duration did not consistently influence speech outcomes. SDS, speech discrimination scores; CI, cochlear implants.

No significant correlation was observed between preoperative SDS (with hearing aids) and postoperative SDS (with cochlear implants) (*p* = 0.08, *r* = 0.325; Figure 1A). A non-significant negative correlation was noted between age at assessment and postoperative SDS (*p* = 0.14, *r* = −0.271), suggesting a trend toward lower speech recognition outcomes in older patients (Figure 1B). Additionally, the duration of deafness was not significantly associated with postoperative SDS (*p* = 0.139, *r* = 0.276) (Figure 1C).

### 3.2 Association between preoperative cognitive function and postoperative speech discrimination scores

**Figure 2:**
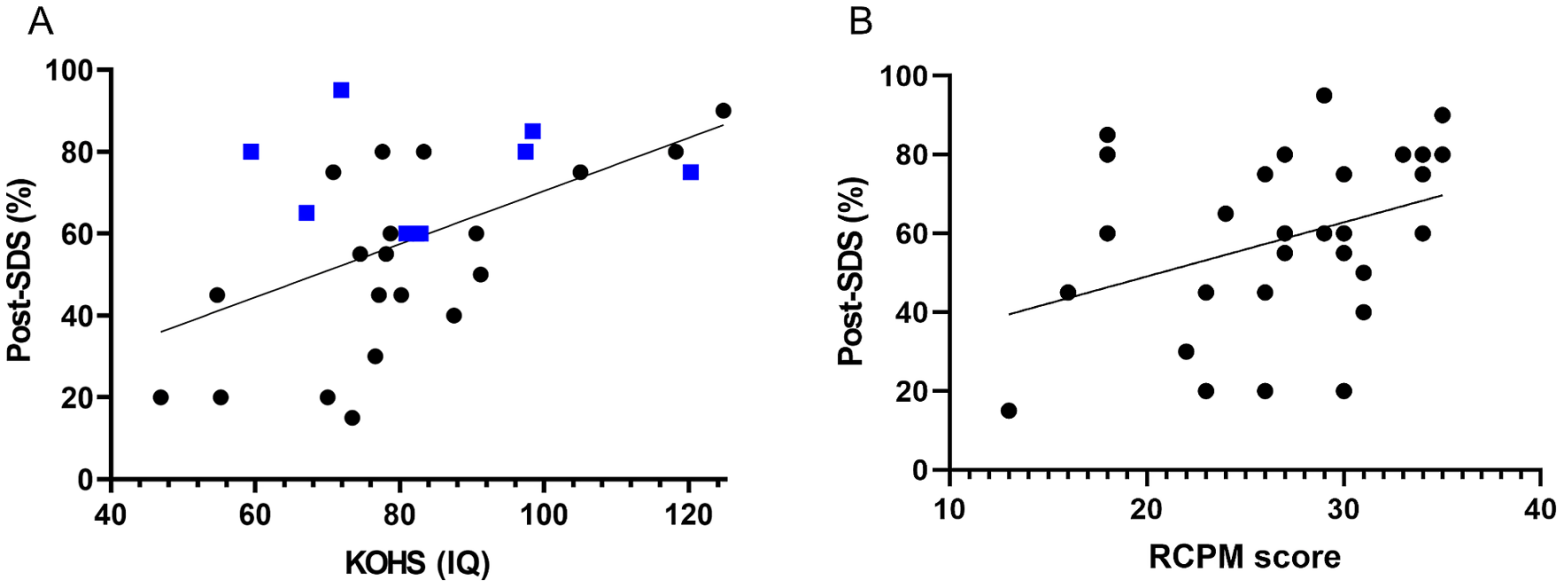
Association between preoperative cognitive function and postoperative speech perception. **(A)** KOHS IQ vs. postoperative SDS: Scatter plot showing a significant positive correlation between preoperative KOHS IQ and postoperative SDS (*p* < 0.01, *r* = 0.538). Higher KOHS scores were associated with better speech perception. Blue markers denote bilateral CI recipients, who generally achieved favorable outcomes, even with lower KOHS scores. **(B)** RCPM scores vs. postoperative SDS: Scatter plot demonstrating a significant positive correlation between preoperative RCPM scores and postoperative SDS (*p* < 0.05, *r* = 0.367), suggesting that stronger nonverbal reasoning abilities were associated with improved speech perception following CI. SDS, speech discrimination scores; CI, cochlear implants; KOHS IQ, Kohs block design test; RCPM, Raven’s colored progressive matrices.

**Figure 3:**
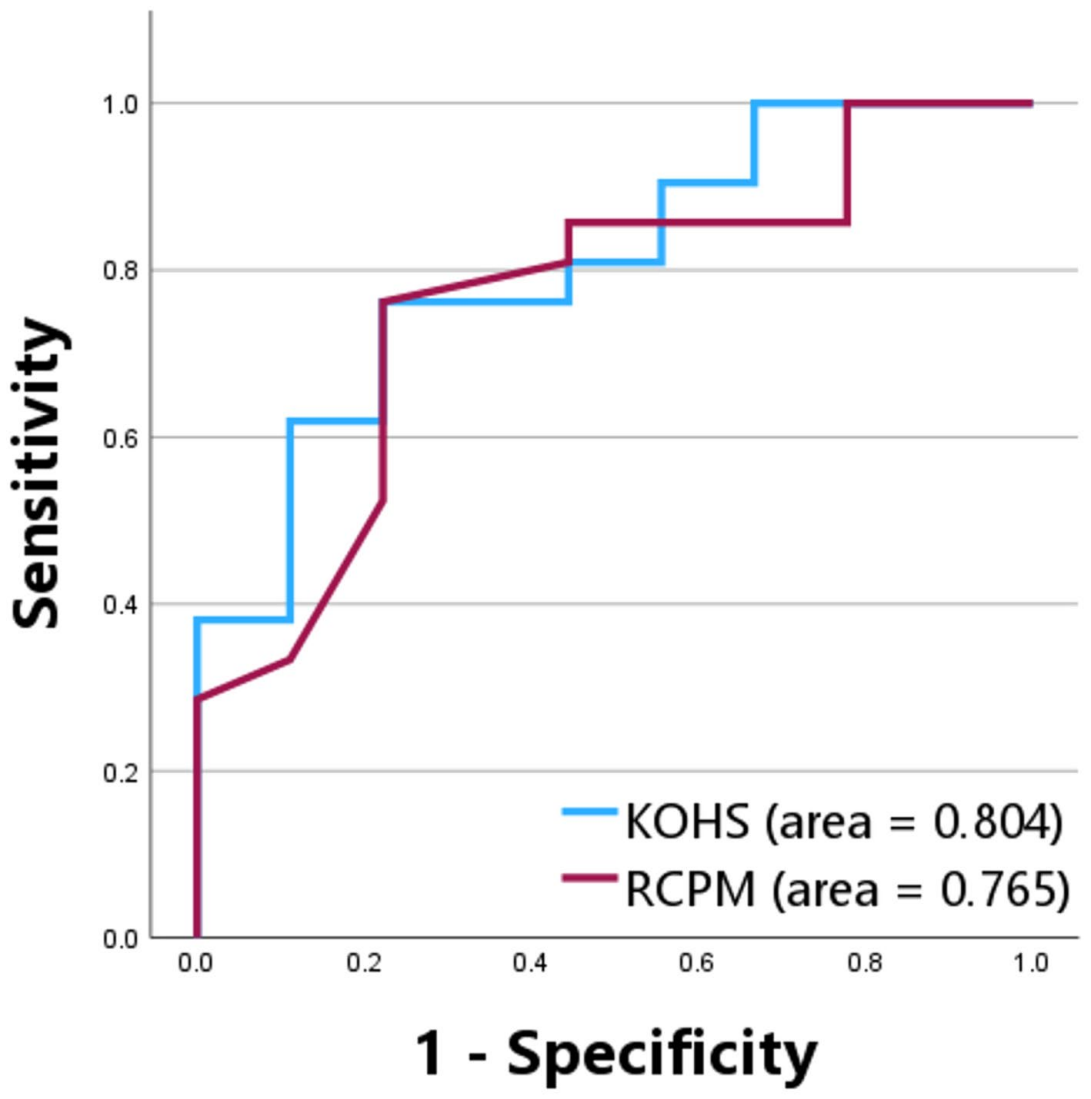
Receiver operating characteristic curves for Kohs block design test and Raven’s colored progressive matrices in predicting postoperative speech discrimination scores. ROC curves evaluating the predictive performance of preoperative KOHS and RCPM scores for postoperative SDS. The AUC was 80.4% for KOHS and 76.5% for RCPM, indicating good discriminative ability. Optimal cutoff values based on Youden’s index were 80.55 for KOHS and 26.5 for RCPM. SDS, speech discrimination scores; KOHS IQ, Kohs block design test; RCPM, Raven’s colored progressive matrices; AUC, area under the curve; ROC, receiver operating characteristic.

Preoperative KOHS IQ scores demonstrated a significant positive correlation with postoperative SDS (*p* < 0.01, *r* = 0.538), indicating that better cognitive performance prior to implantation may predict more favorable speech perception outcomes (Figure 2A). Notably, several bilateral cochlear implant users exhibited high postoperative SDS values despite relatively lower KOHS scores. Similarly, preoperative RCPM scores were significantly correlated with postoperative SDS (*p* < 0.05, *r* = 0.367) (Figure 2B). A moderate correlation was observed between KOHS IQ and RCPM scores (*p* < 0.01, *r* = 0.468). Receiver operating characteristic curve analyses of KOHS and RCPM scores yielded areas under the curve of 80.4 and 76.5%, respectively, with Youden index-derived cutoff values of 80.55 (KOHS) and 26.5 (RCPM) (Figure 3).

### 3.3 Postoperative cognitive function and longitudinal trends

**Figure 4:**
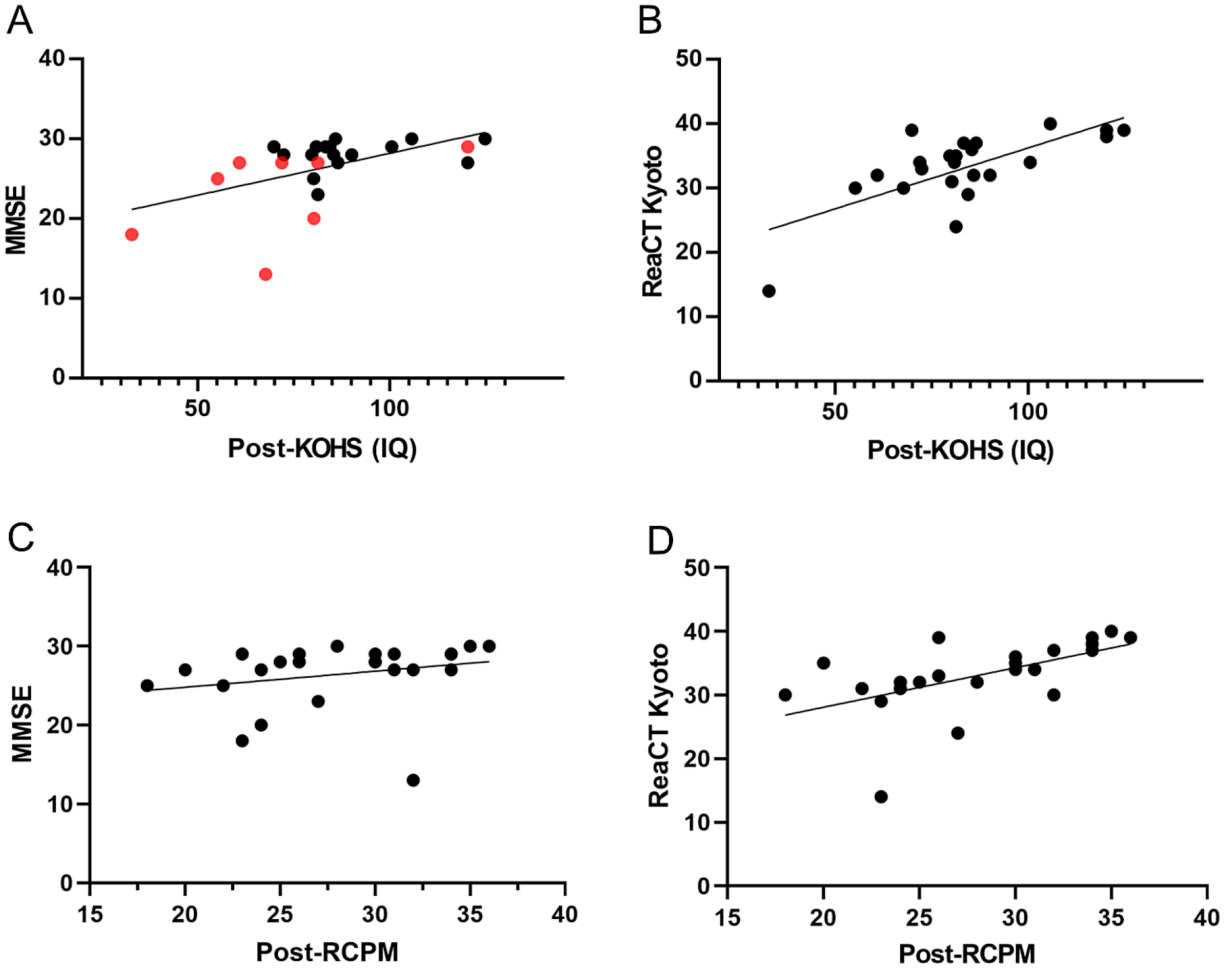
Associations between postoperative cognitive measures. **(A)** Postoperative KOHS IQ vs. MMSE scores: Scatter plot showing a significant positive correlation between KOHS IQ and MMSE scores (*p* < 0.01, *r* = 0.524), indicating that higher visuospatial performance was associated with better global cognitive function. Red markers denote patients with SDS < 50%. **(B)** Postoperative KOHS IQ vs. ReaCT Kyoto scores: Scatter plot revealing a highly significant correlation between KOHS IQ and ReaCT Kyoto scores (*p* < 0.0001, *r* = 0.701), demonstrating strong concordance between these nonverbal cognitive measures. **(C)** Postoperative RCPM vs. MMSE scores: Scatter plot showing no significant correlation between postoperative RCPM and MMSE scores (*p* = 0.246, *r* = 0.246), suggesting that RCPM may capture cognitive domains not assessed by MMSE. **(D)** Postoperative RCPM vs. ReaCT Kyoto scores: Scatter plot demonstrating a significant correlation between RCPM and ReaCT Kyoto scores (*p* < 0.01, *r* = 0.558), supporting their shared focus on nonverbal cognitive function. SDS, speech discrimination scores; KOHS IQ, Kohs block design test; RCPM, Raven’s colored progressive matrices; MMSE, Mini-Mental State Examination; ReaCT, Reading Cognitive Test.

Postoperative KOHS IQ scores showed significant positive correlations with both MMSE (*p* < 0.01, *r* = 0.524) and ReaCT Kyoto scores (*p* < 0.0001, *r* = 0.701) (Figures 4A,B). One patient with an SDS < 50% exhibited an MMSE score of 13 despite a preserved KOHS IQ, suggesting limitations of verbally mediated cognitive assessments in individuals with suboptimal speech perception. While RCPM scores were not significantly correlated with MMSE (*p* = 0.246, *r* = 0.246) (Figure 4C), they were significantly associated with ReaCT Kyoto scores (*p* < 0.01, *r* = 0.558) (Figure 4D).

**Figure 5:**
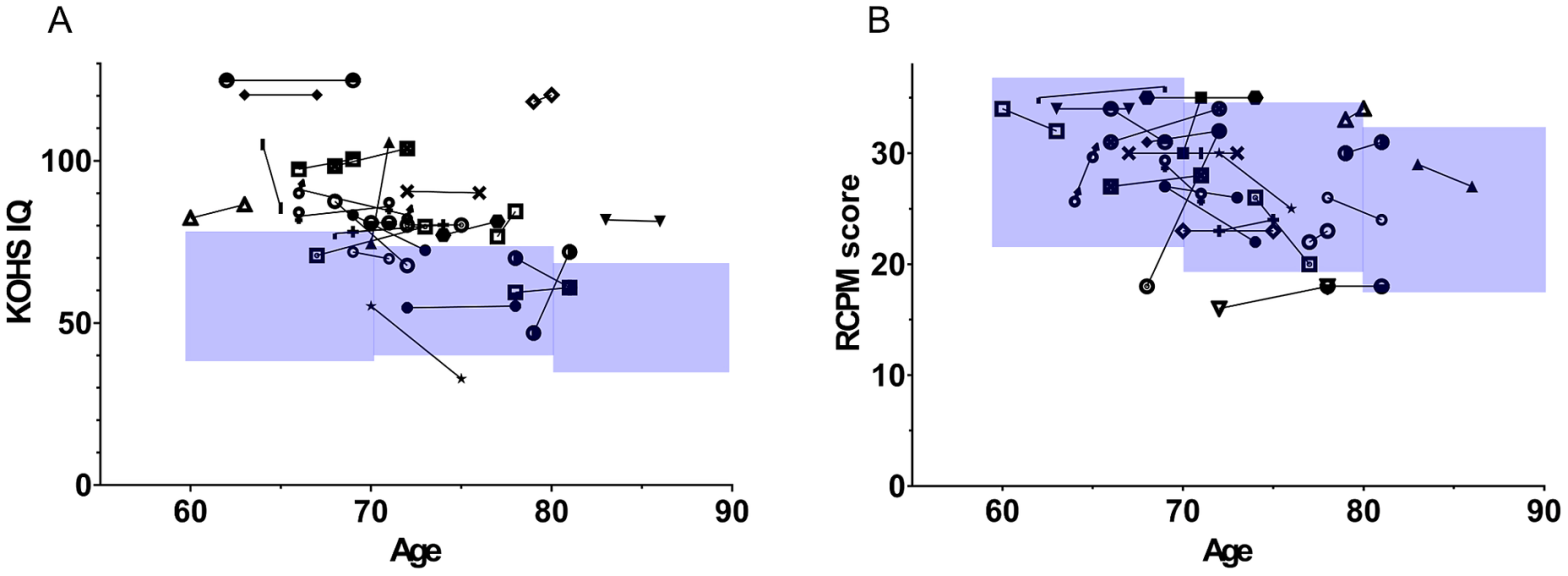
Longitudinal trajectories of Kohs block design test and Raven’s colored progressive matrices scores post-implantation. The figure illustrates age on the x-axis and cognitive function scores on the y-axis. Each symbol represents longitudinal changes in the scores of individual patients. The blue-shaded area in the graph represents the mean ± 1 standard deviation (SD) for older adults in Japan. **(A)** Displays changes in KOHS IQ scores. In most cases, cognitive performance was maintained or improved over time, with only a few cases showing a decline. **(B)** Shows longitudinal changes in RCPM scores. Similar to KOHS IQ results, the majority of patients demonstrated stable or enhanced cognitive function. SDS, speech discrimination scores; CI, cochlear implants; KOHS IQ, Kohs block design test; RCPM, Raven’s colored progressive matrices.

Figure 5A illustrates longitudinal KOHS IQ scores plotted against age. Most patients showed stable or improved scores over time, with only a few cases displaying slight declines. Figure 5B shows similar patterns for RCPM scores, with most patients maintaining or enhancing their cognitive performance postoperatively.

## 4 Discussion

In this study, we examined both the auditory and cognitive outcomes of CI in adults aged ≥ 60 years. With the accelerating pace of global population aging, age-related hearing loss is increasingly prevalent, and CI is emerging as a viable rehabilitative option for individuals with severe-to-profound sensorineural hearing loss. Although cochlear implant outcomes in younger populations are well documented, findings in older adults have shown some variability—likely influenced by age-related changes in cognition and neural plasticity (28).

Several studies have reported improvements in speech perception and quality of life in older CI recipients; however, these findings are not universally consistent. Age-related cognitive decline and reduced neural adaptability have been implicated as potential limiting factors (29, 30). As such, there is growing interest in elucidating CI’s effects on cognition in this demographic.

The interplay between auditory function and cognitive health has gained considerable attention. Hearing loss is now recognized as a modifiable risk factor for dementia (6, 7), highlighting the importance of investigating whether auditory rehabilitation via CI may support cognitive preservation or improvement. In this study, long-term postoperative assessments demonstrated that older CI users generally maintained stable non-verbal cognitive performance, particularly in domains such as spatial reasoning and fluid intelligence. These findings align with a growing body of evidence suggesting that CI may stabilize or even enhance cognitive function in older adults.

An important aspect of this study was the exploration of whether preoperative cognitive function could predict postoperative speech perception. This is clinically relevant for improving patient selection, prognostication, and individualized rehabilitation strategies. These results are consistent with previous findings demonstrating that attention, working memory, and executive control significantly contribute to speech perception, particularly in acoustically challenging settings. For instance, diminished executive function has been associated with reduced speech recognition in noise, independent of peripheral audibility (31). Moreover, individual differences in executive function have been shown to affect selective attention and auditory stream segregation in complex auditory scenes (32).

Nonetheless, the variability in speech outcomes among patients with lower cognitive scores, particularly among bilateral CI users, suggests that cognitive function alone does not fully account for postoperative performance. Additional factors likely include individual differences in neural plasticity, motivation, psychosocial support, and adherence to auditory rehabilitation (33). In a subgroup analysis by cochlear implant manufacturer, a statistically significant difference was observed in postoperative speech discrimination scores. However, given the small sample size and the potential for sampling bias, this difference is unlikely to reflect a true performance disparity between device brands. No significant differences were found in postoperative cognitive outcomes. These findings should be interpreted with caution, and future studies with larger, more balanced cohorts are necessary to determine whether device-specific factors have a meaningful influence on auditory or cognitive outcomes. Furthermore, the inherent advantages of binaural hearing, such as binaural summation, head shadow effect, and spatial release from masking, may enhance speech perception and listening effort, even in individuals with cognitive limitations (34). Bilateral CI has been associated with superior speech understanding in noise, attributable to binaural summation and squelch effects (35). Improvements in spatial hearing in bilateral users may also augment communicative function beyond what is predicted by cognitive performance alone (36).

Altogether, these findings underscore the value of incorporating cognitive assessment into the CI candidacy evaluation process. Integration of cognitive metrics may aid in identifying candidates most likely to benefit from implantation, refining postoperative expectations, and informing personalized rehabilitative strategies. Moreover, recognizing cognitive function as an essential component of CI decision-making may facilitate shared, informed discussions between clinicians, older patients, and their families. In clinical practice, cognitive assessment tools can be used to screen for cognitive vulnerabilities, guide patient counseling, and tailor auditory rehabilitation plans based on individual cognitive profiles. Incorporating these tools at the preoperative stage may help set realistic expectations, optimize post-CI support, and improve long-term outcomes.

We also investigated cognitive changes following CI in this study. Most participants maintained or modestly improved their cognitive performance, with no significant postoperative cognitive decline observed. These findings suggest that CI may support cognitive preservation (37), possibly via improved auditory input. Sarant et al. (38) demonstrated that cochlear implant users showed significant improvements in executive function and working memory, along with stable performance in attention, psychomotor function, and visual learning over a 4.5-year follow-up. In contrast, older adults with untreated hearing loss or normal hearing exhibited cognitive decline over a shorter 3-year period. In line with these findings, our results also suggest that cognitive function remains stable over time following implantation, supporting the view that hearing intervention may serve as a modifiable factor in promoting healthy cognitive aging. These results highlight the importance of incorporating cognitive considerations into preoperative evaluation and long-term care in CI recipients.

Overall, cognitive function tended to remain stable or improve over time following CI. The absence of decline supports the hypothesis that CI may confer a protective effect against age-related cognitive deterioration (37). These findings suggest that the benefits of CI in older adults may not be limited to hearing restoration, but could also be associated with the maintenance of cognitive health and daily functioning. However, further long-term, multidimensional studies—including appropriate control groups—are needed to clarify the potential mechanisms linking auditory rehabilitation and cognitive aging.

### 4.1 Limitations

This study has some limitations. First, the relatively small sample size (*n* = 30) may have limited statistical power and the generalizability of the findings. The cohort included older adults (aged ≥ 60 years) who completed both preoperative and postoperative cognitive assessments; however, some data were missing owing to loss to follow-up, medical deterioration, or death. Specifically, postoperative cognitive data were unavailable for three (KOHS/RCPM) and six patients (MMSE/ReaCT Kyoto), potentially introducing selection bias. Second, heterogeneity in clinical characteristics, such as the wide range in duration of deafness (0.5–56 years), varied etiologies of hearing loss, and differences in laterality of implantation (unilateral vs. bilateral), may have influenced postoperative outcomes. While inclusion criteria, surgical techniques, and device models were standardized to the extent possible, individual variability remains an important consideration in interpreting the results. Third, all implants were from only two manufacturers (Cochlear Corporation and MED-EL), ensuring consistency in device and electrode design but limiting the generalizability of the findings to other devices or future technological advances. Lastly, postoperative cognitive assessments were conducted over a broad timeframe (1–8 years post-implantation), which may have introduced temporal variability, especially given the natural cognitive changes associated with aging. Future studies should incorporate larger, more diverse cohorts, standardized follow-up intervals, and comprehensive outcome measures, spanning cognitive, auditory, and psychosocial domains, to validate and extend these findings. Despite these limitations, the findings of this study provide valuable insights into the cognitive and auditory outcomes of CI in older adults, underscoring the predictive relevance of preoperative cognitive status and the importance of comprehensive preoperative assessment.

### 4.2 Conclusion

This study examined the impact of CI on auditory and cognitive function in older adults. CI significantly improved speech discrimination. Cognitive assessments that are easy to administer may provide helpful insights into predicting cochlear implant outcomes in this population. Additionally, long-term postoperative cognitive function was generally preserved, with the potential for improvement following CI.

## Data Availability

The raw data supporting the conclusions of this article will be made available by the authors without undue reservation.
